# Effect of Garambullo (*Myrtillocactus geometrizans*) Consumption on the Intestinal Microbiota Profile in an Early-Phase Rat Model of Colon Cancer

**DOI:** 10.3390/ijms27021014

**Published:** 2026-01-20

**Authors:** Edelmira Sánchez-Recillas, Enrique Almanza-Aguilera, David Bars-Cortina, Raúl Zamora-Ros, Rosa Iris Godínez-Santillán, Ana Alicia Sánchez-Tusié, Haydé Azeneth Vergara-Castañeda

**Affiliations:** 1 Center for Advanced Biomedical Research, School of Medicine, Autonomous University of Queretaro, Campus Aeropuerto Carretera a Chichimequillas S/N, Ejido Bolaños, Querétaro 76140, Mexico; edelmira.s.r@hotmail.com (E.S.-R.); lic.nutricion.rosairis@gmail.com (R.I.G.-S.); ana.sanchez@uaq.mx (A.A.S.-T.); 2 Unit of Nutrition and Cancer, Cancer Epidemiology Research Program, Catalan Institute of Oncology (ICO), Bellvitge Biomedical Research Institute (IDIBELL), 08908 Barcelona, Spaindbarscortina@gmail.com (D.B.-C.); raulzamoraros@gmail.com (R.Z.-R.); 3 Division of Food and Nutrition Science, Department of Life Sciences, Chalmers University of Technology, 412 96 Gothenburg, Sweden

**Keywords:** colon cancer, garambullo, gut microbiota, bioactive compounds, chemoprevention

## Abstract

Bioactive compounds in food contribute to reducing the risk of developing colon cancer by modulating the gut microbiota. We have recently demonstrated that garambullo (*Myrtillocactus geometrizans*), an endemic fruit of Mexico rich in bioactive compounds, attenuates aberrant crypt foci in an animal model. However, its potential to modulate the gut microbiota is unknown. The main objective of this study was to evaluate whether its consumption modulates colon carcinogenesis by altering the microbiota in an in vivo model induced by azoxymethane and dextran sulfate sodium (AOM/DSS). Fecal samples were collected from twelve male Sprague-Dawley rats and analyzed for microbiota composition after 0, 8, and 16 weeks of treatment with saline (control), AOM/DSS, garambullo (G), or residue of garambullo (RG) with AOM/DSS (G+AOM/DSS and RG+AOM/DSS, respectively). Characterization of the microbiome was based on the conserved region of the 16S rRNA V3-V4 gene, and analyzed by the ZymoBIOMICS’ Targeted Metagenomics Sequencing (Zymo Research) service. In an animal model induced with AOM/DSS for 8 weeks, consumption of G and its residue increased the bacterial genera *Shuttleworthiia*, *Subdoligranulum*, *Lactobacillus*, *Faecalibacterium*, and *Alloprevotella* (*p* < 0.05). Consumption of G and its residue allowed the proliferation of bacteria that produce short-chain fatty acids and are associated with protective mechanisms of the colon.

## 1. Introduction

Colon cancer (CC) is the third most common cancer worldwide and in Mexico. The main cause is an unbalanced diet, characterized by low intake of high-fiber foods and a high consumption of red meat and saturated fats [1]. CC can be prevented by consuming foods rich in fiber and phytochemicals, which have chemoprotective effects in the colon and provide a substrate source for the gut microbiota [2]. One proposed mechanism for protection against inflammatory or carcinogenic processes is modulation of the gut microbiota through the production of secondary metabolites, mainly short-chain fatty acids (SCFAs), derived from bacterial fermentation. These, in turn, promote intestinal homeostasis and modulate signaling pathways involved in tumor invasion processes [3]. Several studies have described the role of the intestinal microbiota in colonic health, with the presence of some bacteria being characterized as beneficial for CC. A greater presence of pathogenic bacteria, such as *Escherichia coli*, *Fusobacterium nucleatum*, and *Bacteroides fragilis*, can produce genotoxic metabolites that damage intestinal cells, promote loss of intestinal barrier function, and create a pro-inflammatory environment [3]. In contrast, a healthy microbiota, as evidenced by an increase in Bacillota or a greater abundance of genera such as *Bifidobacterium* and *Lactobacillus*, favors the production of SCFAs, thus promoting the maintenance of intestinal homeostasis by inducing anti-inflammatory and antiproliferative signals [3,4].

Garambullo (G; *Myrtillocactus geometrizans*) is a fruit endemic to Mexico, rich in bioactive compounds, including a non-digestible fraction mainly composed of dietary fiber and non-soluble (poly)phenols [5,6]. This fraction, also called residue of garambullo (RG), has antioxidant capacity [6]. Previous studies have shown that G is a good substrate source for bacterial fermentation by allowing the generation of SCFAs in an in vitro and in vivo model [5,7]. Likewise, our research group has shown that G consumption exerts a chemoprotective effect by reducing the number of aberrant crypt foci (ACF) in an animal model induced by azoxymethane and dextran sulfate sodium (AOM/DSS) after 16 weeks of treatment [7]. However, it is not clear whether G can modulate the gut microbiota. In the present study, we aimed to analyze changes in the gut microbiota profile in the feces of AOM/DSS-treated rats to help elucidate the mechanisms by which G exerts a protective effect on the colon.

## 2. Results

### 2.1. Diversity of Fecal Microbiota

**Figure 1:**
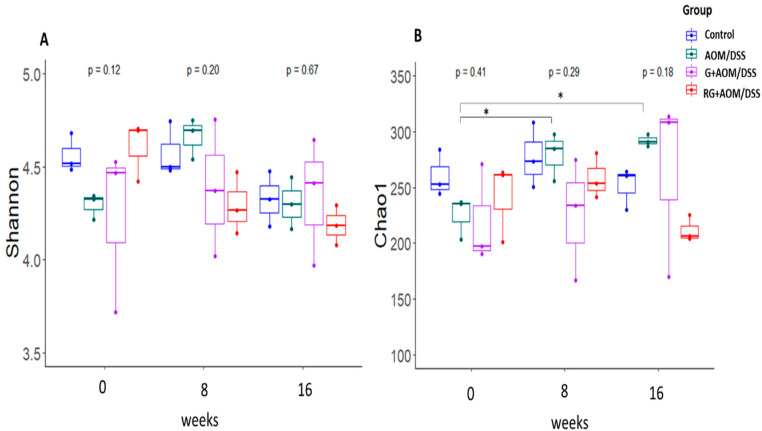
Alpha diversity. (**A**) Shannon index. (**B**) Chao1 index. Data are shown as mean ± standard deviation of three replicates. Statistical differences between and within groups were determined using a Wilcoxon analysis. An asterisk (*) indicates *p* < 0.05. Control, AOM/DSS: Azoxymethane/dextran sodium sulfate, G+AOM/DSS: Garambullo induced with azoxymethane/dextran sodium sulfate, RG+AOM/DSS: residue of garambullo induced with azoxymethane/dextran sodium sulfate.

**Figure 2:**
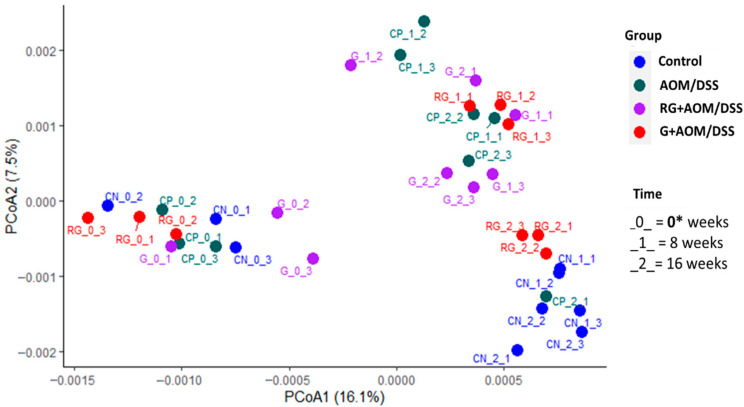
Beta diversity. PCoA (principal coordinate analysis) plot. An asterisk (*) indicates *p* < 0.05. PERMANOVA statistical analysis was used to determine the statistical difference between and within groups. Control, AOM/DSS: Azoxymethane/dextran sodium sulfate, G+AOM/DSS: Garambullo induced with azoxymethane/dextran sodium sulfate, RG+AOM/DSS: residue of garambullo induced with azoxymethane/dextran sodium sulfate.

The α-diversity analysis showed no statistically significant differences in Shannon and Chao1 index values when comparing the four experimental groups within each time window (Figure 1). However, it did when comparing changes over time for each experimental group. The AOM/DSS-induced group showed an increase in α-diversity—as measured by the Chao1 index—at weeks 8 and 16 compared with week 0 (*p* < 0.05). These results suggest that the AOM/DSS group experienced an increase in low-abundance taxa over time compared with the other treatments, given that the Chao1 index is an indicator of low-abundance taxa [8]. Regarding the β-diversity analysis (Figure 2), the microbial composition of the four experimental groups at week 0 showed a statistically significant difference (*p* < 0.05) compared with weeks 8 and 16, suggesting that all experimental groups had a similar gut microbiota composition at the initial time point compared with weeks 8 and 16.

### 2.2. Taxonomical Bacterial Composition

**Figure 3:**
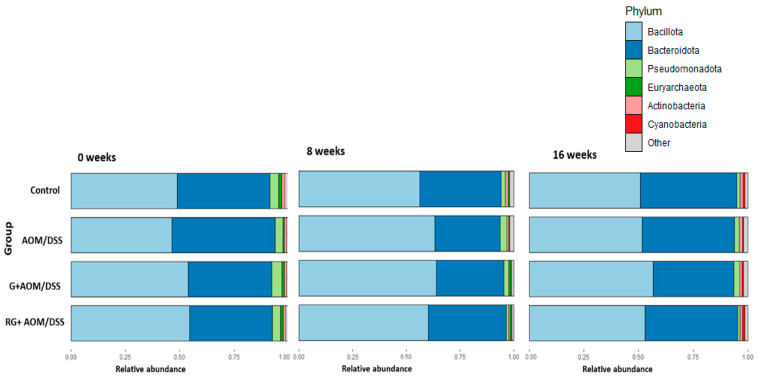
Relative abundance of the four experimental groups. Phylum level. Control, AOM/DSS: Azoxymethane/dextran sodium sulfate, G+AOM/DSS: Garambullo induced with azoxymethane/dextran sodium sulfate, RG+AOM/DSS: residue of garambullo induced with azoxymethane/dextran sodium sulfate.

**Figure 4:**
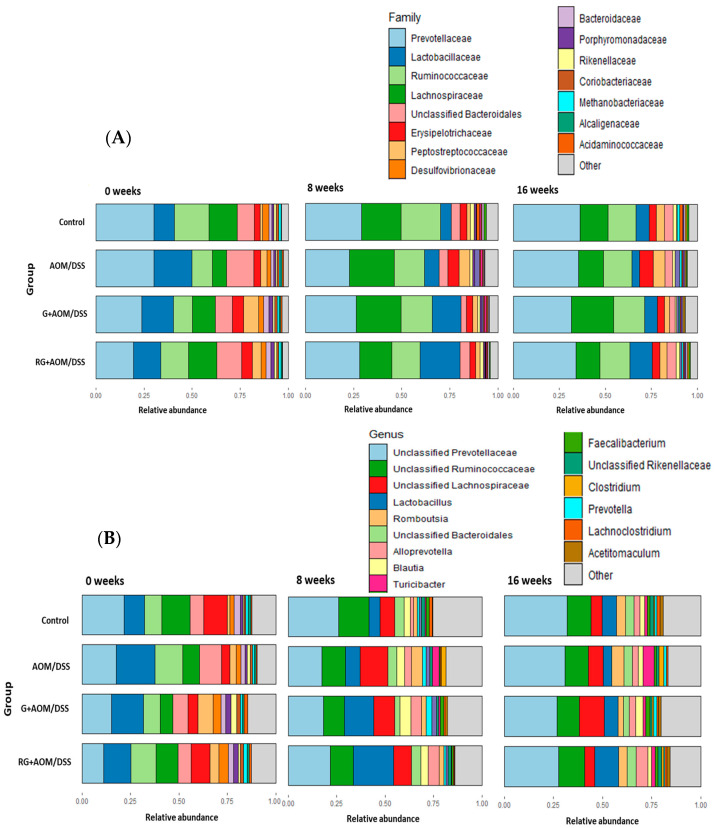
Relative abundance of the four experimental groups. Family level (**A**). Genus level (**B**). Control, AOM/DSS: Azoxymethane/dextran sodium sulfate, G+AOM/DSS: Garambullo induced with azoxymethane/dextran sodium sulfate, RG+AOM/DSS: residue of garambullo induced with azoxymethane/dextran sodium. sulfate.

The relative abundance of the four experimental groups was determined at the phylum, family, genus, and species levels at weeks 0, 8, and 16. The most abundant phyla across the groups were Bacillota, Bacteroidota, Pseudomonadota, Euryarchaeota, Actinobacteria, and Cyanobacteria, regardless of the week of treatment (Figure 3). At the family level, the most abundant in all groups were *Prevotellacae*, *Lactobacillaceae*, *Ruminococcaceae*, and *Lachnospiracea*, which together represented 60% of the total relative abundance (Figure 4A).

After 8 and 16 weeks, a greater abundance of the *Lactobacillaceae* family was observed in the G+AOM/DSS and RG+AOM/DSS groups than in the AOM/DSS and control groups. This family has been described to participate positively in the maintenance of intestinal homeostasis and the production of SCFAs [9]. On the other hand, the results at 8 and 16 weeks show a greater abundance of the *Erysipelotrichaceae* family in the AOM/DSS group than in the control, G+AOM/DSS, and RG+AOM/DSS. The increased abundance of *Erysipelotrichaceae* was described in AOM/DSS-induced mouse models associated with an inflammatory condition [10].

At the genus level, the most abundant among the experimental groups were Unclassified *Prevotellaceae*, Unclassified *Ruminococcaceae*, Unclassified *Lachospiraceae*, *Lactobacillus*, *Romboutsia*, Unclassified *Bacteroidales*, *Alloprevotella*, *Balutia*, *Turicibacter*, *Faecalibacterium*, Unclassified *Rikenellaceae*, *Clostridium*, *Prevotella*, *Lachnoclostridium*, and *Acetitomaculum* (Figure 4B). After 8 and 16 weeks, the G+AOM/DSS and RG+AOM/DSS groups exhibited a higher abundance of *Lactobacillus*, Unclassified *Lachospiraceae*, *Alloprevotella*, *Balutia*, *Faecalibacterium*, and *Prevotella* compared with the AOM/DSS and control groups. On the other hand, the AOM/DSS group showed a greater abundance of the genus *Rombustia*, *Turicibacter*, and *Clostridium* after 8 and 16 weeks compared with the control, G+AOM/DSS, and RG+AOM/DSS groups.

**Figure 5:**
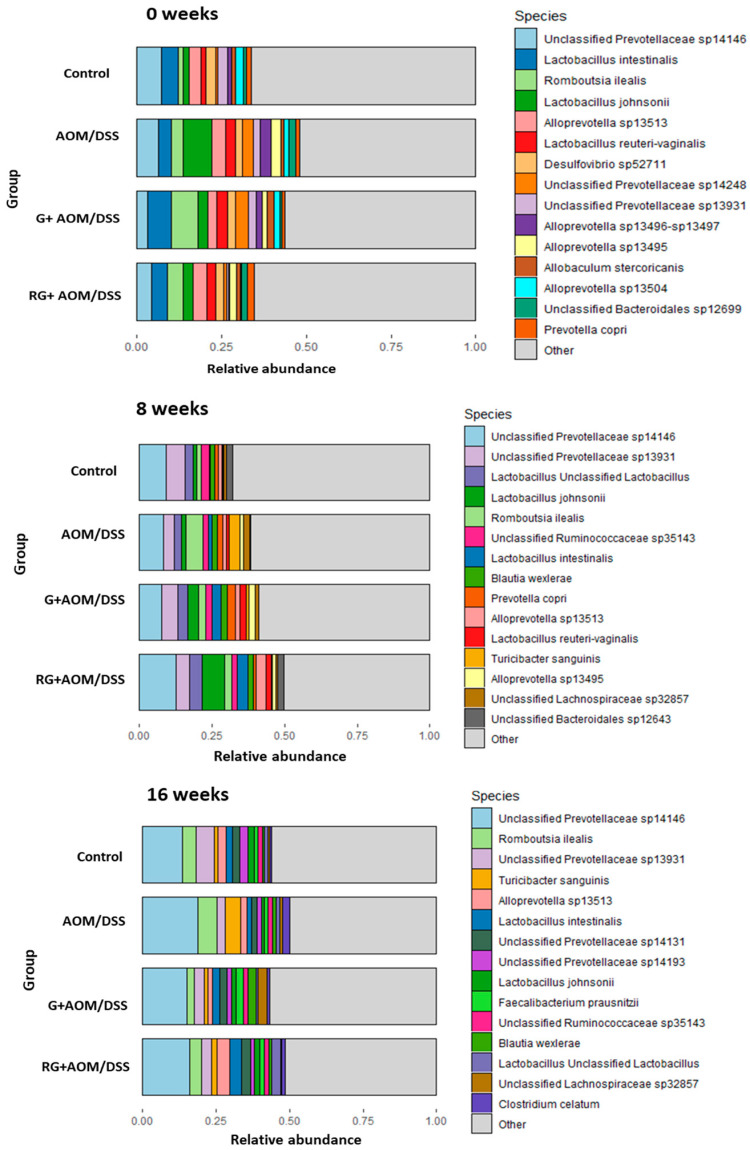
Relative abundance of the four experimental groups. Species level. Control, AOM/DSS: Azoxymethane/dextran sodium sulfate, G+AOM/DSS: Garambullo induced with azoxymethane/dextran sodium sulfate, RG+AOM/DSS: residue of garambullo induced with azoxymethane/dextran sodium sulfate.

At the species level (Figure 5), a higher abundance of *Lactobacillus intestinalis* and *Lactobacillus jhonsoni* was found at weeks 8 and 16 in the G+AOM/DSS and RG+AOM/DSS groups. Similarly, after 16 weeks, a greater abundance of *Faecalibacterium prausnitzii* was observed in the groups fed with G+AOM/DSS and RG+AOM/DSS. On the other hand, at weeks 8 and 16, there was a higher abundance of *Turicibacter sanguinis* in the AOM/DSS group compared with the control, G+AOM/DSS, and RG+AOM/DSS groups.

### 2.3. Bacterial Biomarkers

**Figure 6:**
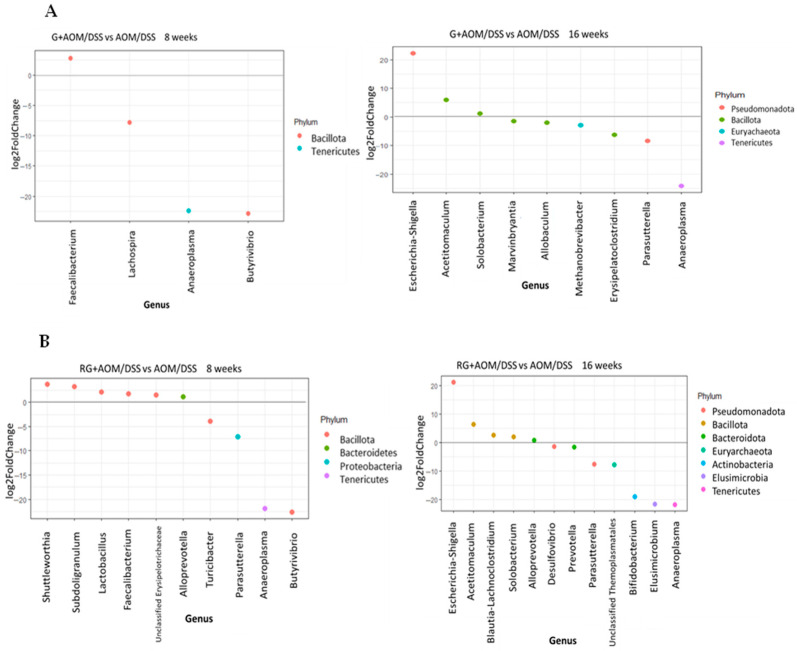
Differential analysis of abundances. The points above the line indicate the bacterial genera that are significantly more abundant in the G+AOM/DSS (**A**) and RG+AOM/DSS groups (**B**) compared with the AOM/DSS group (*p* < 0.05). The analysis was performed using the DESeq2 package. AOM/DSS: Azoxymethane/dextran sodium sulfate, G+AOM/DSS: Garambullo induced with azoxymethane/dextran sodium sulfate, RG+AOM/DSS: residue of garambullo induced with azoxymethane/dextran sodium sulfate.

Figure 6 shows results from the differential abundance analysis (DAA) with the AOM/DSS group as the reference.

The results show that after 8 weeks, the genus *Faecalibacterium* (*p* < 0.05) was more abundant in the G+AOM/DSS and RG+AOM/DSS groups. Similarly, at week 8, the RG+AOM/DSS group showed an increase in the genera *Subdoligranulum*, *Shuttleworthia*, *Alloprevotella*, and *Lactobacillus*. On the other hand, the G+AOM/DSS group exhibited a greater abundance of *Escherichia-Shigella*, *Acetitomaculum*, and *Solobacterium* at week 16, while the RG+AOM/DSS group showed a greater abundance of *Escherichia-Shigella*, *Acetitomaculum*, *Blautia-Lachnoclostridium*, *Subdoligranulum*, and *Alloprevotella*. However, the results show that the consumption of G+AOM/DSS and RG+AOM/DSS at weeks 8 and 16 leads to a decrease in the genera *Turicibacter*, *Desulfovibrio*, *Allobaculum*, *Erysipelatoclostridium*, *Parasutterella*, and *Anaeroplasma*.

## 3. Discussion

Alterations in the colon caused by AOM/DSS increase the permeability of the intestinal mucosa, trigger inflammation, and disrupt the balance of the intestinal microbiota [11]. The aim of this study was, therefore, to investigate whether the consumption of G could exert a protective effect against AOM/DSS induction through changes in the gut microbiota profile.

Treatments with G and RG did not induce changes in α-diversity in response to AOM/DSS induction or over time. There is evidence that supplementation with polyphenols reversed the increase in α-diversity observed in a group of mice induced with AOM/DSS [12]. This may suggest that treatment with G and RG exerts the same effect by reducing the increase in α-diversity observed in the AOM/DSS group over time; as we reported previously, G is rich in a variety of phenolic compounds that resist passage through the gastrointestinal tract and are released in the colon [5,6]. In terms of β-diversity, differences in microbiota composition were observed over time, but not between groups. This is because all four experimental groups had similar bacterial communities at weeks 0, 8, and 16. These results may indicate that the DNA damage caused by AOM, together with the inflammatory changes induced by DSS, causes changes in the composition of bacterial communities [13]. Similarly, the components, such as dietary fiber and polyphenols, contained in G and RG [5,6] interfere with the changes in bacterial communities over time [14].

Analysis of the composition of the microbiota within the four experimental groups showed a higher abundance of the phyla Bacillota and Bacteroidota—the most important groups of bacteria involved in the breakdown of dietary fiber in the colon [15]. The increase in the abundance of Bacillota and Bacteroidota is consistent with reports on rats fed *Ziziphus jujuba* Mill [16], cranberry [17], and *Aronia* berries [18], fruits with nutraceutical characteristics similar to G. The ratio of these two phyla is crucial for maintaining intestinal homeostasis. In addition, animal models have shown that they play an important role in preventing the development of CC [19]. Our results show a higher abundance of Bacillota in the four experimental groups over time. This increase was reported in in vivo models fed with tea polyphenols [20]. However, within this phylum, there are species associated with inflammation and colon cancer, as well as other beneficial butyrate-producing bacteria for the colon, such as the genera *Faecalibacterium*, *Lactobacillus*, and *Roseburia* [21].

The consumption of G+AOM/DSS promoted an increase in the abundance of the genus *Faecalibacterium*, whereas RG+AOM/DSS caused an increase in the abundance of the genera *Subdoligranulum*, *Shuttleworthia*, *Alloprevotella*, *Lactobacillus*, and *Faecalibacterium*. The increase in these bacterial genera could restore or prevent the dysbiosis caused by the inflammatory state in the colon as a result of AOM/DSS induction [22]. The increase in *Lactobacillus*, *Faecalibacterium*, and *Alloprevotella* in the RG+AOM/DSS group could restore intestinal homeostasis by enabling intestinal barrier function, inhibiting the growth of pathogens, reducing the inflammatory state, and preventing the progression of CC [23]. The bacteria of the genus *Alloprevotella* are SCFA producers (acetic, propionic, and butyric acids). These secondary metabolites interfere with the mechanisms of action by regulating the effects of CC progression, reducing the inflammatory state, and restoring intestinal homeostasis through the activation of G-protein coupled receptors (GPCRs) [24]. Additionally, the genus *Alloprevotella* promotes favorable changes in the microbiota profile by increasing the abundance of *Lactobacillus*. This is because they are fiber-degrading bacteria that produce monosaccharides, which are later used by *Lactobacillus* as substrates for fermentation [25]. The increase in *Lactobacillus* abundance may indicate that RG modulates the inflammatory response to AOM/DSS, as *Lactobacillus* is widely recognized as a probiotic. It can reduce the risk of intestinal inflammation by promoting anti-inflammatory signals and activating the immune response through the generation of SCFAs [26]. In turn, it can inhibit the growth of pathogenic bacteria by generating antimicrobial signals in the lumen as it lowers the intestinal pH through lactic and acetic acid production, thus preventing intestinal dysbiosis [19]. *Lactobacillus* can also intervene in antitumor mechanisms. It has been described to protect against the development of CC in AOM/DSS-induced animal models by limiting the activity of β-glucuronidase, which is essential for reactivation of AOM in the colon by hydrolyzing the glucuronic group. It also inhibits the proliferation and apoptosis of cancer cells [27]. The increase in the abundance of *Faecalibacterium* in the groups that consumed G and RG indicates a favorable modulation of the microbiota profile in response to AOM/DSS induction. *Faecalibacterium* has been shown to reduce ACF formation in a rat model induced with AOM [22]. The protection of colon health by *Faecalibacterium* is primarily attributed to butyric acid, as it is one of the major butyrate-producing bacterial genera. It produces butyric acid through the fermentation of dietary fiber, which is present in G, or via a cross-feeding process. Its abundance increases in the presence of *Lactobacillus*, as *Faecalibacterium* metabolizes acetate through the enzyme butyryl-CoA:acetate-CoA transferase, which converts extracellular acetate and intracellular butyryl-CoA into butyrate and acetyl-CoA [28]. This cross-feeding of metabolites could explain the increase in the abundance of acetate-producing bacteria, such as *Alloprevotella* and *Lactobacillus*, together with the increase in the abundance of *Faecalibacterium* observed in our results. As previously mentioned, the increased abundance of *Alloprevotella*, *Lactobacillus*, and *Faecalibacterium*, along with their potential protective effect against AOM/DSS induction, is associated with the effect of SCFAs. SCFAs were not identified in the present study; however, a previous study by Godínez-Santillán et al. (2024) using an AOM/DSS-induced animal model determined SCFA production in the feces of animals that consumed G and berry cactus residue [7]. Therefore, a relationship between changes in the gut microbiota profile and SCFA production could be suggested.

*Faecalibacterium* plays a key role in reducing inflammation, regulating the immune system, maintaining the gut microbiota, and protecting the intestinal barrier. It promotes interleukin (IL)-10 expression, stimulates regulatory T cells, and inhibits pro-inflammatory effector T cells [29]. The butyric acid produced by *Faecalibacterium* inhibits histone deacetylase (HDAC), which reduces NF-kB signaling and the production of pro-inflammatory interleukins [30]. *Faecalibacterium* may also support the restoration of tight junction proteins, such as claudin-2 and zonula occludens 1 (ZO1), which are essential for epithelial integrity and may be disrupted during carcinogenic processes [31]. Furthermore, it plays a role in maintaining intestinal homeostasis. It also helps maintain intestinal homeostasis by regulating colonic oxygen (O_2_) levels. AOM/DSS induction leads to an inflammatory state that causes persistent oxidative stress, altering luminal O_2_ levels and promoting dysbiosis. *Faecalibacterium* may protect the colonic environment through the action of flavin reductase, an enzyme that transfers electrons to O_2_, thereby helping preserve anaerobic conditions and limiting the growth of pathogenic bacteria sensitive to low O_2_ levels. This allows the maintenance of the anaerobic environment and prevents the growth of opportunistic pathogenic bacteria that cannot survive under low O_2_ conditions [32]. Thus, the increase in *Lactobacillus* and *Faecalibacterium* observed in this study could be related to the protective effect of G, previously reported by our research group. In that earlier study, 16 weeks of G consumption reduced the development and multiplicity of ACF in an AOM/DSS-induced animal model [7]. However, it is important to note that analyzing inflammatory biomarkers is crucial, as they allow the protective effect of G to be related to the induction of AOM/DSS, a perspective that will be examined later in this project.

The results show that consumption of G and RG at weeks 8 and 16 promotes a decrease in the genera *Turicibacter*, *Desulfovibrio*, *Allobaculum*, *Erysipelatoclostridium*, *Parasutterella*, and *Anaeroplasma*. The genus *Allobaculum* is positively correlated with inflammatory gastrointestinal disorders and inflammatory biomarkers such as tumor necrosis factor alpha (TNF-α) in both human and animal models [33]. *Anaeroplasma* is elevated in AOM/DSS-induced animal models and associated with immunosuppressive mechanisms in patients with inflammatory bowel disease (IBD) [34]. *Turicibacter* and *Parasutterella* are opportunistic pathogens whose increased abundance has been reported in CC models induced by AOM/DSS and DSS colitis [35,36]. The increase in their abundance has been linked to alterations in the intestinal barrier, damage to the mucus layer, and the triggering of immune responses, leading to inflammation [37]. *Erysipelatoclostridium* has been identified in an early stage of CC in humans [15]. *Desulfovibrio* can degrade sulfated mucin in the intestine, allowing it to colonize the intestine. Its proliferation promotes IL-6 secretion, which triggers the inflammatory response. Moreover, the metabolite produces hydrogen sulfide (H_2_S), penetrates cell membranes unhindered, and blocks the butyrate oxidation pathway in epithelial cells, which has a toxic effect on these cells [38]. Compounds such as quercetin have been reported to decrease the abundance of the genus *Desulfovibrio*, suggesting that the quercetin contained in G may modulate this genus [39].

The observed changes in the microbiota profile in the G+AOM/DSS and RG+AOM/DSS groups could be due to secondary metabolites derived from the fermentation of dietary fiber and polyphenols present in both food matrices. Dietary fiber is a substrate for bacterial fermentation by *Lactobacillus*, *Alloprevotella*, and *Faecalibacterium* [37]. Similarly, the compounds contained in G, such as phenolic acids, quercetin, and betalains, have been described to promote the growth of *Faecalibacterium* and *Lactobacillus* [10,40]. Onali et al. (2025) reported that the consumption of berries (200 g daily) by healthy individuals on a diet high in red meat may protect against the development of CC. Berry intake resulted in secondary metabolites derived from polyphenols and an increased abundance of the *Faecalibacterium* genus, both of which were associated with the inhibition of colon adenocarcinoma cell viability (HCA-7 and Caco-2). The preventive potential of berry consumption against CC was linked to a higher intake of dietary fiber, vitamin C, manganese, and polyphenols present in berries [41]. In general, polyphenols (supplementary Table S1) are known to inhibit the growth or increase of bacteria by interfering with bacterial DNA and RNA synthesis, altering bacterial cell membrane function, and impairing bacterial biofilm formation [12]. Additionally, polyphenols can regulate gut health by acting differently on Gram-positive and Gram-negative bacteria through their distinct interactions with their cell walls, and exert an antimicrobial effect [42]. These mechanisms could therefore explain the effect of the bioactive compounds contained in G on the modulation of the intestinal microbiota, as reported in the results.

## 4. Materials and Methods

### 4.1. Collection and Treatment of Garambullo

The G was collected in the community of Garabatillo, Celaya, in the state of Guanajuato (Mexico) during June 2021. The fruits were selected according to their ripeness, characterized by an intense purple color and being plump to the touch. After harvesting, the G was washed under running water (without scrubbing), freeze-dried, ground, and stored in sealed bags at −80 °C. The RG, which refers to the non-digestible fiber fraction along with the non-soluble bioactive compounds, was obtained by methanol-water (30:70 *v*/*v*) extraction of the freeze-dried G. It was centrifuged at room temperature at 10,000× *g* for 10 min and filtered with Whatman paper (0.20 µm). The supernatant was discarded, and the RG was obtained and stored in sealed bags at −80 °C.

### 4.2. Animals and Reagents

Twelve four-week-old male Sprague-Dawley rats were used. The rats were housed in the vivarium of the Institute of Neurobiology of the National Autonomous University of Mexico (UNAM). They were kept at an ambient temperature of 24 ± 2 °C with a 12/12-h light/dark cycle, and provided with water and food ad libitum. All procedures were carried out in accordance with NOM-062-200-1999. This project was approved by the Bioethics Committee of the Faculty of Medicine of the Autonomous University of Queretaro (No. 13778). The carcinogen used was azoxymethane (AOM (No. A5486, Sigma-Aldrich, Louis, MO, USA), 10 mg/kg body weight, dissolved in 1 mL of physiological solution), and DSS (No. 42867, Sigma-Aldrich), 2% dissolved in water, was used as a tumor promoter.

### 4.3. Experimental Design

**Figure 7:**
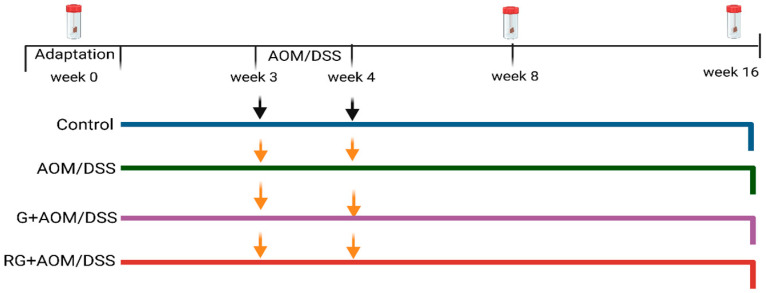
In vivo experimental design. Control: basal diet and subcutaneous saline injection. AOM: Azoxymethane. DSS: dextran sodium sulfate. G: garambullo. RG: residue of garambullo. Feces samples were collected at weeks 0, 8, and 16. Orange arrows indicate induction with AOM/DSS. Black arrows indicate induction with saline solution.

One week after acclimatization (Figure 7), rats were randomly assigned to one of the following treatment groups (n = 3 per group): (1) Control, basal diet (RodentLab Chow 5001), and subcutaneous saline injection; (2) AOM/DSS: basal diet plus a subcutaneous injection of AOM (10 mg/kg) once a week for two weeks (3rd and 4th week), plus DSS 2% for 7 days; (3) G+AOM/DSS: lyophilized G (5 g/kg body weight) and induction with AOM/DSS and basal diet; and (4) RG: a dose of 5 g/kg body weight administered once daily in pellet form during the experimental period (16 weeks), induced with AOM/DSS (RG+AOM/DSS) and basal diet. The dose was determined to be safe according to a previous report by Reynoso et al. (2015) [43,44].

### 4.4. DNA Extraction and 16S rRNA Sequencing

Fecal samples were collected at three time points: at the beginning (week 0), in the middle (week 8), and at the end (week 16). To avoid contamination, rats belonging to the same group were placed in individual plastic boxes without sawdust for 15 min prior to fecal sampling. Once collected, fecal samples (200–300 mg) were immediately placed in DNA/RNA Shield fecal sample tubes (R1101, Zymo Research Corp., Irvine, CA, USA) and stored at −80 °C until their analysis. Fecal DNA was extracted using the ZymoBIOMICS DNA Miniprep Kit (D4300, Zymo Research Corp, Irvine, CA, USA), quality assessed using a NanoDrop 2000 spectrophotometer (Thermo Fisher, Waltham, MA, USA), and stored at −80 °C until microbiota data analysis.

A total of three samples per group, each consisting of a pooled DNA sample from three rats, were diluted to 20 µg/µL with sterile water. Processing and analysis were performed using the ZymoBIOMICS Targeted Metagenomic Sequencing Service (Zymo Research, Irvine, CA, USA). Libraries were prepared with the NGS Quick-16S™ (Zymo Research, Irvine, CA, USA) Kit and V3-V4 primers via real-time PCR. Libraries were quantified by qPCR fluorescence, pooled based on molarity, and purified using Select-a-Size DNA Clean & Concentrator™ (Zymo Research, Irvine, CA, USA). Final quantification was performed with TapeStation^®^ (Agilent Technologies, Santa Clara, CA, USA) and Qubit^®^ (Thermo Fisher Scientific, Waltham, MA, USA). The ZymoBIOMICS microbial community standard was used as a positive control. Sequencing was conducted on an Illumina^®^ MiSeq™ platform (v3 kit, 600 cycles) with 10% PhiX. Unique sequence variants were identified and chimeras removed using DADA2 [45], and taxonomic assignment was performed with UCLUST (QIIME v1.9). Bioinformatic analysis utilized the proprietary Zymo Research database. Relative abundance was determined by quantitative PCR using a plasmid DNA standard curve containing the 16S gene, and the same primers used for library preparation. Copy number quantification was calculated based on 2 μL of DNA input, assuming a genome size of 4.64 × 10^6^ bp (*Escherichia coli*).

### 4.5. Intestinal Microbiota Analysis

The analysis of the intestinal microbiota was performed with R and RStudio (version 4.2.3). α-diversity analysis measured species richness and was calculated using the Shannon and Chao1 indexes. β-diversity was used to determine differences in microbial community composition structure among groups and was analyzed using the compositional data analysis (CODA) method. The distance between microbial compositions was calculated using Bray-Curtis analysis. Differential abundance analysis (DAA) identified statistically significant differences between comparisons at each time point at the genus level. This was calculated using the *DESeq2* R-based package, using a generalized linear model for the comparison of individual taxa among experimental groups [46].

### 4.6. Statistical Analysis

Statistically significant differences between groups were determined using the Wilcoxon test for α-diversity, and a permutational multivariate analysis of variance (PERMANOVA) was applied for β-diversity. Statistical significance was assumed at *p* < 0.05 for all tests. Results are expressed as mean ± standard deviation (SD) of three rats per group. The analysis was performed using RStudio version 4.2.3.

## 5. Conclusions

Changes in bacterial abundance across the four experimental groups over time were observed at the phylum, family, genus, and species levels. Significant changes in bacterial abundance within the experimental groups were described at the genus level. Treatment with G and RG after 8 weeks increased the abundance of bacterial genera associated with protective mechanisms of the colon, including the genera *Lactobacillus*, *Faecalibacterium*, and *Alloprevotella*. Similarly, the consumption of G after 8 weeks proved to be a good substrate for the proliferation of bacteria of the genus *Faecalibacterium*. Overall, the current results suggest that G and its residues may be a substrate for the proliferation of beneficial bacteria that counteract induced colon carcinogenesis in an in vivo model.

## Data Availability

The original contributions presented in this study are included in the article. Further inquiries can be directed to the corresponding authors.

## Supplementary Materials

The following supporting information can be downloaded at: https://www.mdpi.com/article/10.3390/ijms27021014/s1.
