# Supplementary material for: Effect of Garambullo (Myrtillocactus geometrizans) Consumption on the Intestinal Microbiota Profile in an Early-Phase Rat Model of Colon Cancer
Source: Int J Mol Sci. 2026 Jan 20;27(2):1014. doi: 10.3390/ijms27021014 (PMC12842398; doi:10.3390/ijms27021014)
Supplement: Supplementary file 1 [file ijms-27-01014-s001.zip › ijms-3988546-supplementary.pdf]

Table S1. Compounds reported to be present in garambullo.

**Bioactive compounds**	**Chemical structure**	**Bacteria associated**
Kaempferol [17,20,18,6]	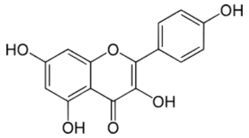	*Clostridium*
Quercetin [17,21,18,36,6]	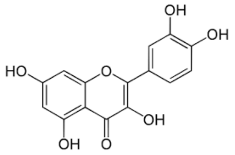	*Bacteroides*<br>*Enterococcus*<br>*Butirivibrio*<br>*Clastridium*
Betalains [17,21,6,39]	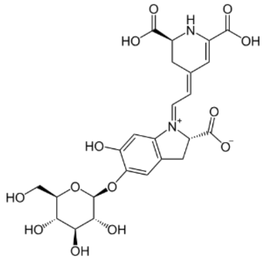	*Firmicutes*<br>*Proteobacterias*<br>*Firmicutes*<br>*Lachnospirace*<br>*Blautia*<br>*Lactobacillus*<br>*Akkermansia muciniphela*<br>*Rombustia*<br>*Ruminoccocace*
Caffeic acid [6,36]	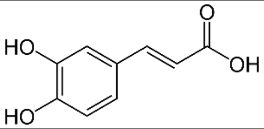	*Lactobacillus*
Ferulic acid [21,6]	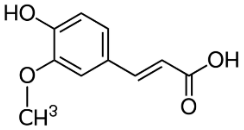	*Bifidobacterium*<br>*Lactobacillus*<br>*Clastridium*
Phenolic acids (hydroxycinnamic acid) [17,21,6,36]	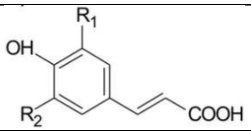	*Bifidobacterium*<br>*Lactobacillus*
